# School Attendance and Public Health

**Published:** 1907-08-24

**Authors:** 


### School Attendance and Public Health.

Long ago there was an outcry against the grants to State-supported schools being made dependent upon the children passing certain examinations, because it was felt that this method tended to over-pressure and consequent injury to the children. In deference to this cry, the grant was made to depend upon the average attendance. But the result of this has been, as was recently pointed out by Dr. Conolly, medical officer of health of Wood Green, that teachers press children to come to school even when they are not in a fit state of health. There is a general notion among those who have to do with education that a high average attendance is a proof of efficiency on the part of the teachers. That irregular attendance means imperfect education may be granted, but that is no reason for keeping up the average attendance by insisting on the presence of sickly children, who may be in the incubation period of infectious diseases, and are in any case unlikely to benefit by what they are taught.

Another effect of the pecuniary importance of a high average attendance is that when the appearance of an epidemic keeps a considerable number of children away from school, the teachers press for the closing of the entire school. Partly, no doubt, this is due to the fear of infection spreading through the school; though as the pupils who are thus set free play for the most part in the streets in company with school-fellows, many of whom are either sickening for the disease or convalescent from it, it may be doubted if the preventive value of the closing is great. But also, the teachers prefer to show a smaller number of sessions with a large average attendance at each, than a greater number with a lower average, because the amount of the grant is reckoned on the actual sessions and average attendances. Thus this method of settling the grant is not more favourable to education than the old way, nor more beneficial to public health. The ideal method of fixing what a school has earned from the Government is doubtless difficult to find, but at least it is desirable that teachers should not be made to feel that the securing of a high average attendance is an important feature of their work.

